# The Elderhood Mystique

**DOI:** 10.1093/geroni/igab046.1916

**Published:** 2021-12-17

**Authors:** Kate de Medeiros

**Affiliations:** Miami University, Oxford, Ohio, United States

## Abstract

While elderhood recognizes untapped potential and continued growth and creativity in later life, it also risks becoming a dismissive label that positions older people as different, otherworldly, and mysterious. By analyzing the concept of elderhood and similar movements (e.g., sageing, croning, eldering) in popular and academic literature, paying close attention to how elderhood is defined and framed, I found that elderhood has a role in some religious and cultural practices. However, newer approaches to elderhood have emerged from middle aged writers who imagine an idealized role in later life – the elderhood mystique. Often grounded in introspective passivity and selflessness, elderhood parallels Kathleen Woodward’s depiction of wisdom as a disempowering label that discourages activism and resistance by older people. Subsequently, elderhood and wisdom risk becoming new forms of othering or exclusion. Overall, findings underscore the importance of critical analysis of age-related terms, regardless of how positive they seem.

